# Preoperative and Intraoperative Risk Factors for Conversion of Laparoscopic Cholecystectomy to Open Cholecystectomy: A Systematic Review of 30 Studies

**DOI:** 10.7759/cureus.47774

**Published:** 2023-10-27

**Authors:** Xinlin Chin, Sachini Mallika Arachchige, Jane Orbell-Smith, Arkadiusz P Wysocki

**Affiliations:** 1 General Surgery, Mackay Base Hospital, Mackay, AUS; 2 Medicine, Griffith University, Birtinya, AUS; 3 Medicine and Dentistry, James Cook University, Mackay, AUS; 4 Surgery, Canberra Hospital, Australian Capital Territory (ACT) Health, Canberra, AUS; 5 General Surgery, Caboolture Hospital, Caboolture, AUS; 6 General Surgery, Logan Hospital, Meadowbrook, AUS

**Keywords:** a systematic review, intraoperative risk factors, preoperative risk factors, acute cholecystitis, cholecystitis, gallbladder, risk factors for conversion, open cholecystectomy, lap converted to open cholecystectomy, laparoscopic cholecystectomy (lc)

## Abstract

This systematic review aims to review articles that evaluate the risk of conversion from laparoscopic to open cholecystectomy and to analyze the identified preoperative and intraoperative risk factors. The bibliographic databases CINAHL, Cochrane, Embase, Medline, and PubMed were searched according to the Preferred Reporting Items for Systematic Reviews and Meta-Analyses (PRISMA) guidelines. Only English-language retrospective studies and systematic reviews with more than 200 patients were included. The time of publication was limited from 2012 to 2022. Our systematic review identified 30 studies with a total of 108,472 patients. Of those, 92,765 cholecystectomies were commenced laparoscopically and 5,477 were converted to open cholecystectomy (5.90%). The rate of conversion ranges from 2.50% to 50%. Older males with acute cholecystitis, previous abdominal surgery, symptom duration of more than 72 hours, previous history of acute cholecystitis, C-reactive protein (CRP) value of more than 76 mg/L, diabetes, and obesity are significant preoperative risk factors for conversion from laparoscopic to open cholecystectomy. Significant intraoperative risk factors for conversion include gallbladder inflammation, adhesions, anatomic difficulty, Nassar scale of Grades 3 to 4, Conversion from Laparoscopic to Open Cholecystectomy (CLOC) score of more than 6 and 10-point gallbladder operative scoring system (G10) score more than 3.

## Introduction and background

Symptomatic gallstones are usually effectively managed with cholecystectomy [1]. There have been ongoing discussions about whether laparoscopic cholecystectomy (LC) or open cholecystectomy (OC) is the better approach for acute cholecystitis; however, based on the Tokyo Guidelines 2018, LC is preferred for the treatment of acute cholecystitis [1,2]. The rate of conversion from LC to OC ranges from 1% to 24% [1,3,4]. Bile duct injury is a feared complication as LC (two to five times higher in LC compared to OC) has become more widely performed and the clinical prognosis of vasculobiliary injury is poor [5]. Hence, identifying the risk factors for conversion helps to improve surgical planning [1].

The aim of this systematic review is to analyze the published evidence with a view to determining preoperative characteristics predictive of conversion. The secondary aim is to identify intraoperative characteristics highly predictive of conversion to enable surgeons to make the decision to convert early.

## Review

Method

This systematic review and meta-analysis were conducted according to Preferred Reporting Items for Systematic Reviews and Meta-Analysis (PRISMA). The research question driving the review was: What are the preoperative and intraoperative risk factors for conversion of LC to OC?

Eligibility Criteria 

The literature search strategy was created based on the following eligibility criteria: English language articles. The inclusion criteria for the systematic review was reported conversion from laparoscopic to OC. The time of publication was limited from 2012 to 2022. 

Search Strategy 

The search strategy was created as ((Cholecystitis OR (Biliary tract disease*) OR (Gall bladder)) AND ((Laparoscopic cholecystectomy) AND (Open Cholecystectomy)) AND (Conversion OR Risk)) and undertaken by the professional health librarian author in February 2022. The search strategy was modified to fit Embase.

Databases Interrogated

The bibliographic databases CINAHL, Cochrane, Embase, Medline, and PubMed were searched. Duplicates were removed and refinement of the topic was undertaken by the librarian, after which the title and abstract of the records were independently screened by the authors.

Data Collection and Data Items 

The studies included in the systematic review were analyzed to identify conversion risk factors. The identified variables were then extracted from the included studies. The collected study data were number of patients, year of publication, and statistical methods. The extracted outcome variables included age, gender, weight or Body Mass Index (BMI), prior abdominal surgeries, prior biliary admissions, symptom duration, body temperature, American Society of Anesthesiologists (ASA) classification, comorbidities, acute or elective surgery, diagnosis, preoperative Endoscopic Retrograde Cholangio-Pancreatography (ERCP), preoperative liver function test, preoperative white cell count, ultrasound and Magnetic Resonance Cholangio-Pancreatography (MRCP) findings, use of intraoperative cholangiography (IOC), intraoperative scoring (Nassar and 10-point gallbladder operative scoring system G10) and intraoperative findings.

Results

**Figure 1:**
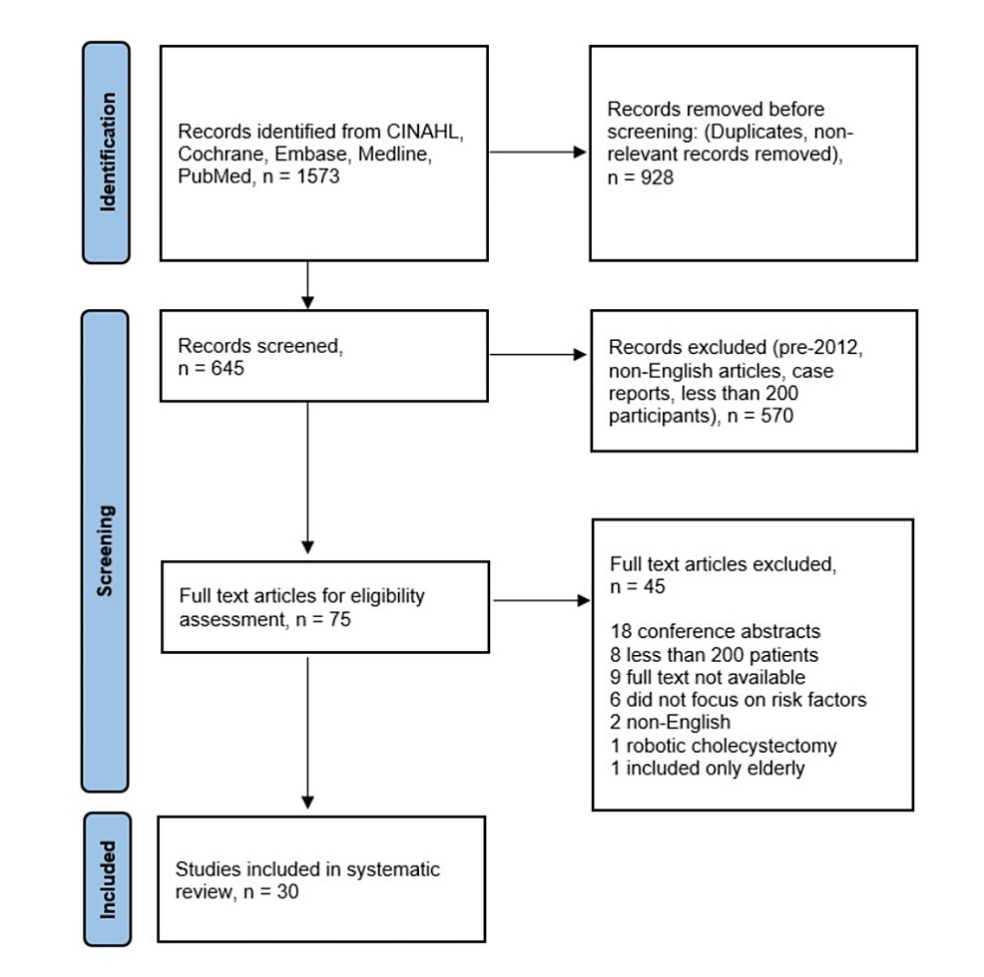
Preferred Reporting Items for Systematic reviews and Meta-Analyses (PRISMA) flow chart showing methods for article selection

One thousand five hundred seventy-three papers were initially retrieved in response to the search strategy. Of those, 928 papers that were duplicates and non-relevant to the research topic were removed, leaving 645 papers for title and abstract screening. Of those, 75 papers were identified for full-text assessment. A further 45 papers were excluded, leaving 30 papers for inclusion in the systematic review (Figure 1).

Our systematic review included 30 studies with a total of 108,472 patients [1,4,6-33]. 92,765 patients underwent LC and 5,477 were converted to OC (Table 1). The 30 studies consist of 26 retrospective analyses, three prospective cohort studies and one systematic review.

**Table 1:** Preoperative and intraoperative risk factors for conversion of laparoscopic to open cholecystectomy. ALP: Alkaline phosphatase; ALT: Alanine transaminase; BMI: Body mass index; CLOC: Conversion from Laparoscopic to Open Cholecystectomy; CRP: C-reactive protein; CT: Computed tomography; ERCP: Endoscopic retrograde cholangiopancreatography; G10: 10-point gallbladder operative scoring system; GGT: Gamma-glutamyl transferase; LC: Laparoscopic cholecystectomy; WCC: White cell count

Author, Year	Total	LC cases	Converted cases	Conversion rate	Preoperative risk factors (P value)	Intraoperative risk factors (P value)
Botaitis, 2012 [26]	2412	2412	150	6.20%	Age >65 (0.016), Male (0.053), Diabetes (0.051), Previous upper abdominal surgeries (0.009), Previous abdominal surgeries (0.001), Temperature >37.5 (0.008), Aspartate transaminase >60 U/L (0.017), Alanine transaminase >60 U/L (<0.001)	
Hasbahceci, 2012 [28]	1557	1557	39	2.50%	Age > 55 (< 0.01), Male (<0 .001), Previous acute cholecystitis attacks (<0 .001), Emergency admission due to acute cholecystitis (<0 .001)	
Le, 2012 [30]	3413	3371	86	2.60%		Inflammation, Adhesions, Anatomic difficulty (not specified)
Lee, 2012 [31]	346	100	41	11.90%	Age >60 (0.0002), Male (0.003), Previous upper abdominal surgeries (0.0008), Acute cholecystitis (0.0028), Gallbladder wall thickness >3mm on ultrasound (0.0073)	
Cwik, 2013 [27]	5596	4104	130	24% (acute cholecystitis) and 3.4% (uncomplicated gallbladder disease)	BMI > 25-30 kg/m^2^ (<0.05), Delay of surgery >72 hours from time of admission (<0.05), Ultrasound findings: Gallbladder wall >5mm (<0.000001), Blurring of Calot's triangle anatomy (0.003), Pericholecystic exudate (0.002), Pericholecystic abscess (0.0001)	Difficult identification of the cystic duct, Severe inflammatory or post-operative adhesions (not specified)
Wevers, 2013 [4]	261	261	62	24%	Age > 65 (0.02), CRP >165 mg/L (<0.001)	Inability to reach the critical view of safety due to inflammatory changes (not specified)
Asai, 2014 [6]	225	225	29	12.90%	Symptom duration> 72 hours (0.0004), CRP > 11.5 mg/dL (0.0041)	
Kim, 2014 [29]	183	183	30	17%	Age >60 (0.007), Male (0.014), CT findings: Pericholecystic fluid (<0.001)	
Licciardello, 2014 [32]	414	414	33	7.90%	Age >65 years (0.036), Acute cholecystitis (0.001)	
Yajima, 2014 [7]	407	407	47	11.60%	Male (0.042), Acute cholecystitis (<0.001)	
Goonawardena, 2015 [8]	732	732	47	6.4%%	Previous upper abdominal surgery (0.0041), Obesity/BMI > 30 kg/m^2^ (0.00011), Ultrasound findings: Choledocholithiasis (0.00013), Gallbladder wall width in mm with no specific cut-offs (<0.000001), Impacted stone at neck of gallbladder (0.0053)	
Sippey, 2015 [9]	7242	7242	436	6%	Age >60 years (0.0015), Male (0.005), BMI > 30 kg/m^2^ (0.0001), Preoperative ALP (0.0005), WCC with no specific cut-offs (0.0001), Albumin with no specific cut-offs (0.0001)	
Beksac, 2016 [10]	1335	1231	104	7.70%	Age >55 years (0.001), Male (0.005), ALP >80 IU/L (0.026), Previous abdominal surgeries (0.011), Choledocholithiasis (not specified), Ultrasound findings of acute cholecystitis (not specified)	
Ishizuka, 2016 [11]	461	461	25	5.40%	Preoperative hypoalbuminaemia <3.8g/dL (0.022), GGT <20 IU/L or >20 IU/L (0.038), Platelet <27 or >27 × 104/mm3 (0.039)	
Sutcliffe, 2016 [12]	8820	8820	297	3.40%	CLOC score >6 (not specified), Nassar grade 3-4 (not specified)	
Terho, 2016 [13]	373	373	84	22.50%	CRP > 150 mg/L (<0.001), Age >65 years (0.023), Diabetes (0.045)	Gallbladder gangrene (<0.001), Abscess formation (<0.001)
Hu, 2017 [1]	57,503	53,669	3500	6.52%	Age >65, Male, High body mass index, Acute cholecystitis (not specified)	
Utsumi, 2017 [15]	236	236	19	8%	Previous upper abdominal surgeries (0.001), Emergency laparoscopic cholecystectomy (0.007), Acute cholecystitis (0.028), Ultrasound findings: Pericholecystic fluid (0.005)	
Bouassida, 2017 [14]	493	493	56	11.40%	Male (0.012), Diabetes (0.019), Total bilirubin level (0.045), Tokyo Guidelines 13: moderate acute cholecystitis (<0.001)	
Spence, 2017 [33]	2521	2521	248	9. 80	Concurrent biliary tract disease (0.03)	
Chavez, 2018 [16]	1386	1386	14	16 (Emergency surgery) versus 2.6 (Elective)	Emergency surgery (0.05), Chronic cholecystitis associated to previous biliary colics (0.001), ALT elevation (0.002) Ultrasound findings: Common bile duct dilatation >6mm (0.001), Hepatomegaly (0.05)	
Coffin, 2018 [17]	2810	2810	139	4.95%	Age 65 and above (<0.001), Male (<0.001), Emergency admission (<0.001), Urgent admission (<0.001)	
Sugrue, 2019 [18]	504	504	71	14%		G10 score of 3 (<0.0001), Buried gallbladder (0.018), Impacted stone >1 cm (0.0257), Bile/pus outside gallbladder (0.0046), Fistula (0.0066)
Kara, 2019 [22]	2076	1950	115	5.90%	Age (0.009), Male (<0.001), Acute cholecystitis (not specified)	Inadequate dissection of Calot triangle due to fibrotic adhesions (not specified), Adhesions due to previous surgery (not specified)
Bouasidda, 2020 [19]	556	556	75	13.48%	CRP >76 mg/L (0.0001)	
Hirohata, 2020 [20]	395	324	33	9.20%	Hypoalbuminaemia <3.2g/dL (0.043), CT findings: Duodenal oedema (0.014)	
Jang, 2020 [21]	581	581	113	19%	Obesity (BMI >30) (0.04), Previous abdominal surgeries (0.03), Prolonged prothrombin time (0.03), CT findings: Absence of gallbladder wall enhancement (0.03), Gallstone in gallbladder infundibulum (0.04), Inflammation of hepatic pedicle (0.04)	
Warchalows, 2020 [23]	527	527	263	50%	Age >60 (0.0000), Male (0.0000), Diabetes (0.0211), Neurological disease (0.0384)	
Abraham, 2021 [24]	4013	4013	167	4.20%	Age > 65 years (<0.001), Male (<0.001), Previous ERCP (<0.001), Previous upper abdominal surgery (<0.001), History of acute cholecystitis (<0.001)	
Sapmaz, 2021 [25]	1294	1294	41	3.16%	Age >65 (<0.001), Male (<0.001), Acute cholecystitis (<0.001)	

Risk Factors for Conversion From LC to OC

Age: Age was assessed in 20 studies [1,6,7,9,10,13,14,17,19,21-26,28-32]. Fifteen studies found age to be a significant risk factor with most studies using age over 60 or 65 years as the definition of older age [1,4,9,12,13,17,22-26,28,29,31,32]. 11.9% of patients aged 65 and above underwent conversion from LC to OC compared to only 3.3% of patients below the age of 65 years [17]. Two studies reported age more than 55 to be a risk factor where the conversion risk was 4.66 times higher [10,28].

Male: Fourteen out of 20 studies found male gender to be a significant risk factor [1,4,6,7,9,10,12-14,17,21-26,28,29,31,32]. The risk of conversion was increased by four-fold [1]. Five studies reported that male gender was not a significant risk factor [4,6,13,21,32].

BMI: Obesity (BMI ≥30 kg/m^2^) and BMI ranging from less than 24.9 to more than 30 kg/m^2 ^were evaluated in 11 studies [1,8-10,12,16,17,21,26-28​​​]. Obesity and BMI greater than 25 to 30 kg/m^2 ^were significant risk factors for conversion [1,8,9,16,21,26,27].

Prior abdominal surgeries: Fifteen studies evaluated prior abdominal surgeries (upper and lower abdominal) as a risk factor with eight studies finding it significant [1,7,8,10,13,15,21,22,24,26-29,31,32]. Cwik et al. reported a 25% increase in the risk of conversion with prior upper abdominal surgeries while Beksac et al. reported the conversion risk with previous abdominal surgeries was 2.54 times higher [10,27]. The risk of conversion from LC to OC in patients who previously underwent lower abdominal surgeries was slightly elevated at 4.8% but this increased to 18.8% with upper abdominal surgeries [24]. Utsumi also identified previous distal gastrectomy as a significant risk factor due to the formation of adhesions [15].

ASA class: ASA class was evaluated in four studies, where three demonstrated a positive association with conversion [1,4,9,12]. One of the papers reported an ASA score of more than 3 was a significant risk factor while Sippey et al. reported that 11.9% of patients with ASA Class 4 or 5 underwent conversion [1,9].

Diabetes: Nine studies assessed diabetes as a risk factor and three of them found this to be a significant risk factor [1,9,13,17,19,21,23,26,31]. There was no description regarding the type of diabetes (insulin-dependent or other) studied in these papers. Diabetic patients have a higher risk of developing gangrenous cholecystitis hence increasing the risk of conversion by 1.9-fold [1,13,14,23,26]. However, a few studies reported that diabetes was not a significant risk factor for conversion [17,21,31]. The latter two papers were more recent, i.e., published in or after 2018.

Comorbidities: Two out of eight studies found medical comorbidities to be a significant risk factor while four studies demonstrated a specific positive association between hypertension, hyponatremia, respiratory and hematological disorders [1,6,7,9,14,15,23,32]. Cardiovascular patients on antiplatelet agents or anticoagulants and patients with neurological diseases (5.26 times higher) were at increased risk of conversion [15,23].

Prior biliary admissions: The one study that evaluated prior biliary admissions (acute cholecystitis, pancreatitis) found it to be a significant risk factor. Patients who had prior hospitalizations for acute cholecystitis had higher conversion rates (14%) due to chronic inflammatory changes of the gallbladder [26].

Prior acute cholecystitis attacks: One study evaluated prior acute cholecystitis attacks as a risk factor and found it to be significant. In that study, 38.5% of the patients who underwent conversion had previous episodes of acute cholecystitis [28].

Emergency surgery: Three studies evaluated emergency surgery as a risk factor, and all found it to be significant. Patients who underwent emergency LC had a higher rate of conversion compared to patients who underwent elective LC (16% versus 2.6%) [1,15,16].

Acute cholecystitis: Ten out of 18 studies found acute cholecystitis to be a significant risk factor for conversion [1,4,6,7,9-11,15,16,22-28,31,33]. Acute cholecystitis was diagnosed clinically in five out of the 10 studies and histologically in three studies [7,11,15,22,24,25,28,31]. The remaining studies did not provide an explanation regarding the diagnosis of acute cholecystitis. The conversion risk between Grade 1 (21.8%) and Grade 2 (21%) acute cholecystitis was similar [15]. The rate of conversion was 1.5% for patients with cholelithiasis and ranged from 12.3% to 41% in acute cholecystitis [24,25]. 

Gallbladder wall thickness: Gallbladder wall thickness on ultrasound was assessed in six studies and was found to be significant in two studies [1,8,10,13,26,27​​​​​​]. Gallbladder wall of more than 5 mm was associated with a 31% conversion rate in one study [27].

Symptom duration: Seven studies evaluated symptom duration and three studies found symptom duration of more than 72 hours to be a significant risk factor [1,4,6,9,14,19,27]. More than 50% of the patients who had symptoms for at least four days required conversion to OC. Their conversion rates were five-fold higher compared to patients who had shorter symptom duration [27].

Preoperative endoscopic retrograde cholangio-pancreatography (ERCP): One out of five studies that assessed the use of preoperative ERCP found it to be a significant risk factor [1,8,12,24,32]. 8.8% of patients who had preoperative ERCP underwent conversion from LC to OC due to biliary obstruction [24]. The timeframe between ERCP and cholecystectomy was not mentioned in the papers.

Liver function test: Ten out of 12 studies showed that elevated alkaline phosphatase, alanine aminotransaminase, total bilirubin, and reduced albumin were significant risk factors for conversion [1,6,9-11,16,19-21,26,27,32]. An elevated total bilirubin increased the risk of conversion by three-fold [1].

C-reactive protein (CRP): Five studies reported elevated CRP to be a significant risk factor [4,6,11,13,19]. Bouassida reported CRP as an independent risk factor for conversion and that the optimum CRP cut-off value for predicting conversion was 76 mg/L [19].

White cell count: Three out of seven studies found elevated white cell count to be a significant risk factor [4,6,9-11,26,32]. Botaitis reported a white cell count of more than 9 x 10^9^/L to be significant, but the rate of conversion was not documented in any of the studies [26].

Nassar scale: Grade three or four on the Nassar operative difficulty scale was a significant risk factor for conversion (Table 2) [12,34].

**Table 2:** Nassar operative difficulty grading scale.

Grade	Gallbladder	Cystic pedicle	Adhesions
1	Floppy, non-adherent	Thin and clear	Simple up to the neck/Hartmann’s pouch
2	Mucocoele, packed with stones	Fat-laden	Simple up to the body
3	Deep fossa, acute cholecystitis, contracted, fibrosis, Hartman's adherent to common bile duct, impaction	Abnormal anatomy or cystic duct-short, dilated, or obscured	Dense up to fundus; involving hepatic flexure or duodenum
4	Completely obscured, empyema, gangrene, mass	Impossible to clarify	Dense, fibrosis, wrapping the gall bladder, duodenum or hepatic flexure difficult to separate
5	Grade 4 findings plus Mirizzi Syndrome Type 2 or higher; cholecystocutaneous, cholecystodueodenal, cholecystocolic fistula		

G10 Cholecystitis severity score (10-point gallbladder operative scoring system): A cut-off score of 3 was a significant predictor for conversion in the only study to look at this (Table 3) [18]. The factors forming the G10 score are gallbladder adhesions, completely buried gallbladder, distended or contracted gallbladder, inability to grasp without decompression, stone >1 cm impacted in Hartmann’s pouch, BMI > 30kg/m^2^, adhesion from prior surgery, free bile or pus outside the gallbladder, presence of a fistula.

**Table 3:** G10 cholecystitis severity score.

Cholecystitis severity	Score
Appearance	
Adhesions	1
Adhesions > 50% but gallbladder buried	2
Completely buried gallbladder	3 (max)
Distension or contraction	
Distended gallbladder or contracted shrilled gallbladder	1
Inability to grasp without decompression	1
Stone > 1 cm impacted in Hartmann’s pouch	1
Access	
BMI > 30	1
Adhesions from previous surgery limiting surgery	1
Sepsis and complications	
Free bile or pus outside the gallbladder	1
Fistula	1
Total possible	10

Conversion from laparoscopic to open cholecystectomy (CLOC) score: Patients with CLOC scores more than 6 are at higher risk of conversion (7.1% versus 1.2%) (Table 4) [12]. Components of CLOC score are age, male gender, indication, ASA class, thickened gallbladder wall, and dilated bile duct.

**Table 4:** Components of CLOC score. ASA: American Society of Anesthesiologists physical status classification system, CLOC: Conversion From Laparoscopic to Open Cholecystectomy

	Points
Age	
<30	0
30–39	2
40–69	3
70+	5
Gender	
Female	0
Male	1
Indication	
Colic/Pancreatitis	0
Cholecystitis	2
Common bile duct stone	3
ASA	
1	0
2	2
3+	3
Gallbladder wall	
Normal	0
Thick walled	1
Common bile duct diameter	
Normal	0
Dilated	1

Discussion

We found that older age, male gender, previous abdominal surgeries, acute cholecystitis, symptom duration of more than 72 hours, previous history of acute cholecystitis, CRP value, CLOC score more than 6, G10 cholecystitis severity score more than 3, diabetes, obesity, emergency LC, and urgent admissions are significant risk factors for conversion of LC to OC. Complications after OC are more frequent and more severe than after LC [35].

An understanding of these variables may be applied in several ways. Patients with multiple risk factors should be counseled about the high risk of conversion and its associated morbidity. The surgeon faced with a patient with these features could opt to start the cholecystectomy open or defer a time of day when senior assistance is available. Identification of unfavorable features intraoperatively may prompt a change in technique (e.g., subtotal cholecystectomy) or early conversion to open. A liberal policy of subtotal cholecystectomy may not avoid bile duct injury [36]. The chance of unplanned laparotomy in patients aged above 60 increases by 1.05 times per year [23]. There is a three-to-five-fold increase in the risk of conversion from LC to OC in patients aged above 65 possibly secondary to the longer history of gallbladder disease, masked symptoms leading to delayed hospital presentation [1,13]. Gallstone disease is two to three times more common in females but the risk of conversion from LC to OC is higher in men [1]. It is postulated that males are more prone to developing acute cholecystitis and tend to delay seeking help hence leading to delayed hospital presentation with more severe gallbladder disease at the time of operation [1,25].

Acute cholecystitis is a significant risk factor that causes a five to 14 times increase in the conversion of LC to OC [1,7,11,15,22,24,25,28,31]. Severe inflammation features in acute cholecystitis such as thickened and gangrenous friable gallbladder wall, short cystic duct, gallbladder scarring, and its dense adherence to the common bile duct in acute cholecystitis lead to difficult anatomy identification and dissection hence conversion to OC [1,22].

Obesity is a significant and independent risk factor for conversion of LC to OC due to higher pneumoperitoneum requirements, difficulty with liver retraction due to steatotic stiffness, difficult trocar placement and laparoscopic instruments manipulation, and heavy fat infiltration in the hepatocystic triangle hindering adequate anatomy visualization [1,8,9,21,26,27,37]. Previous abdominal surgeries may cause peritoneal adhesions which affect access to the gallbladder and the hepatocystic triangle [1,27].

CRP is a better risk conversion predictor than white cell counts due to its significant increase in patients with gangrenous cholecystitis [19]. CRP is also an indicator of mortality and post-operative complications [6]. A pre-operative albumin level of less than 32 g/L is an independent risk factor for conversion from LC to OC [11,20]. Hypoalbuminemia reflects chronic malnutrition and chronic inflammation in advanced gallbladder disease which contribute to difficult dissection [9,20]. Other pre-operative laboratory findings that are associated with a higher risk of conversion include elevation of total bilirubin (possibly secondary to Mirizzi syndrome) and alkaline phosphatase levels [1,9,10,14,27].

Ultrasound findings in patients requiring conversion from LC to OC included pericholecystic fluid (42%), thickened gallbladder wall of >5 mm (31%), common bile duct dilatation of more than 6mm, impacted stone at gallbladder neck, contracted gallbladder and gallbladder abscess [8,13,16,27]. Every millimeter measured above the 6mm common bile duct was associated with a 40% increase in risk of conversion [16]. Duodenal edema shown on computed tomography imaging was an independent risk factor as it reflects severe inflammation that extends beyond the gallbladder wall [20].

Common intraoperative risk factors for conversion from LC to OC seen in 75% to 93% cases include severe inflammation (35%) and adhesions affecting anatomy visualization (28%), which lead to difficult dissection of the hepatocystic triangle [1,8,13,21,30]. Other causes include difficult pneumoperitoneum establishment, difficult common bile duct stone retrieval, spilled stones, common bile duct injury, choledochoduodenal fistula, biliary peritonitis secondary to ruptured gallbladder, gangrenous cholecystitis, uncontrolled bleeding, and atypical anatomy secondary to an aberrant biliary duct [8, 21]. Careful dissection is required to prevent bile duct injury in Type 1 Mirizzi syndrome due to the presence of adhesions and inflammatory changes in the hepatocystic triangle [1].

Strengths

Only papers with more than 200 patients were included to improve the quality of the review. Excluding papers prior to 2012 ensures conclusions are based on contemporaneous data. A previous paper included only studies with more than 300 patients and no restrictions were made regarding the time of publication [1]. Another strength is that this systematic review analyses both preoperative and intraoperative risk factors including intraoperative grading systems which are predictors of conversion to OC.

Limitations

Only English-language articles were included. Non-English articles may be available but not identified or included.

## Conclusions

This systemic review revealed older age, male gender, previous abdominal surgeries, acute cholecystitis, symptom duration of more than 72 hours, previous history of acute cholecystitis, diabetes, obesity, emergency LC, and CRP value >76 mg/L to be significant preoperative risk factors for conversion from LC to OC. Significant intraoperative risk factors include a Nassar scale of Grade 3 to 4, CLOC score of more than 6, and G10 cholecystitis severity score of more than 3. Awareness of these risk factors helps to identify patients who are at higher risk of conversion, guide surgeons in their approach to cholecystectomy, and may inform early conversion to OC hence reducing the risk of prolonged surgery. Preoperative discussion of these factors will ensure patients understand they have an increased risk of conversion.
